# What Is the Current Knowledge About Sublay/Retro-Rectus Repair of Incisional Hernias?

**DOI:** 10.3389/fsurg.2018.00047

**Published:** 2018-08-13

**Authors:** Ferdinand Köckerling, Christine Schug-Pass, Hubert Scheuerlein

**Affiliations:** ^1^Department of Surgery and Center for Minimally Invasive Surgery, Academic Teaching Hospital of Charité Medical School, Vivantes Hospital, Berlin, Germany; ^2^Department of General and Visceral Surgery, St. Vinzenz Hospital, Paderborn, Germany

**Keywords:** incisional hernia, sublay, mesh, retro-rectus repair, systematic (literature) review

## Abstract

**Introduction:** There continues to be very little agreement among experts on the precise treatment strategy for incisional hernias. That is the conclusion drawn from the very limited scientific evidence available on the repair of incisional hernias. The present review now aims to critically assess the data available on the sublay/retro-rectus technique for repair of incisional hernia.

**Materials and Methods:** A systematic search of the literature was performed in May 2018 using Medline, PubMed, and the Cochrane Library. This article is based on 77 publications.

**Results:** The number of available RCTs that permit evaluation of the role of the sublay/retro-rectus technique in the repair of only incisional hernia is very small. The existing data suggest that the sublay/retro-rectus technique has disadvantages compared with the laparoscopic IPOM technique for repair of incisional hernia, but in that respect has advantages over all other open techniques. However, the few existing studies provide only a limited level of evidence for assessment purposes.

**Conclusion:** Further RCTs based on a standardized technique are urgently needed for evaluation of the role of the sublay/retro-rectus incisional hernia repair technique.

## Introduction

Numerous guidelines, meta-analyses and systematic reviews explore the best possible surgical treatment for ventral and incisional hernias (1–26). Nonetheless, there is very little agreement among experts on the precise treatment strategy (27). The reasons for that are no doubt manifold. One systematic review of the prospective randomized studies and reviews in the treatment of ventral and incisional hernias found only a limited evidence base for determining the best treatment options for patients despite the large number of patients with ventral and incisional hernias and the high frequency of repair (28). Another analysis of the literature on elective surgery of ventral and incisional hernias identified inconsistencies in reporting of peri- and postoperative variables and poor definition of variables (29). A further problem is the pooling of primary ventral hernias and incisional hernias in many studies, meta-analyses and systematic reviews (30–33). Several studies have demonstrated that there is a significant difference between the outcomes for primary abdominal wall hernias and incisional hernias, hence these hernia entities should not be pooled (30–33). Against that background the present review aims to identify which data are available on the sublay/retro-rectus operation to demonstrate that this is the best open technique for repair of only incisional hernia. In terms of nomenclature the terms “sublay” and “retro-rectus” are intended as equivalent designations (34).

## Materials and methods

**Figure 1:**
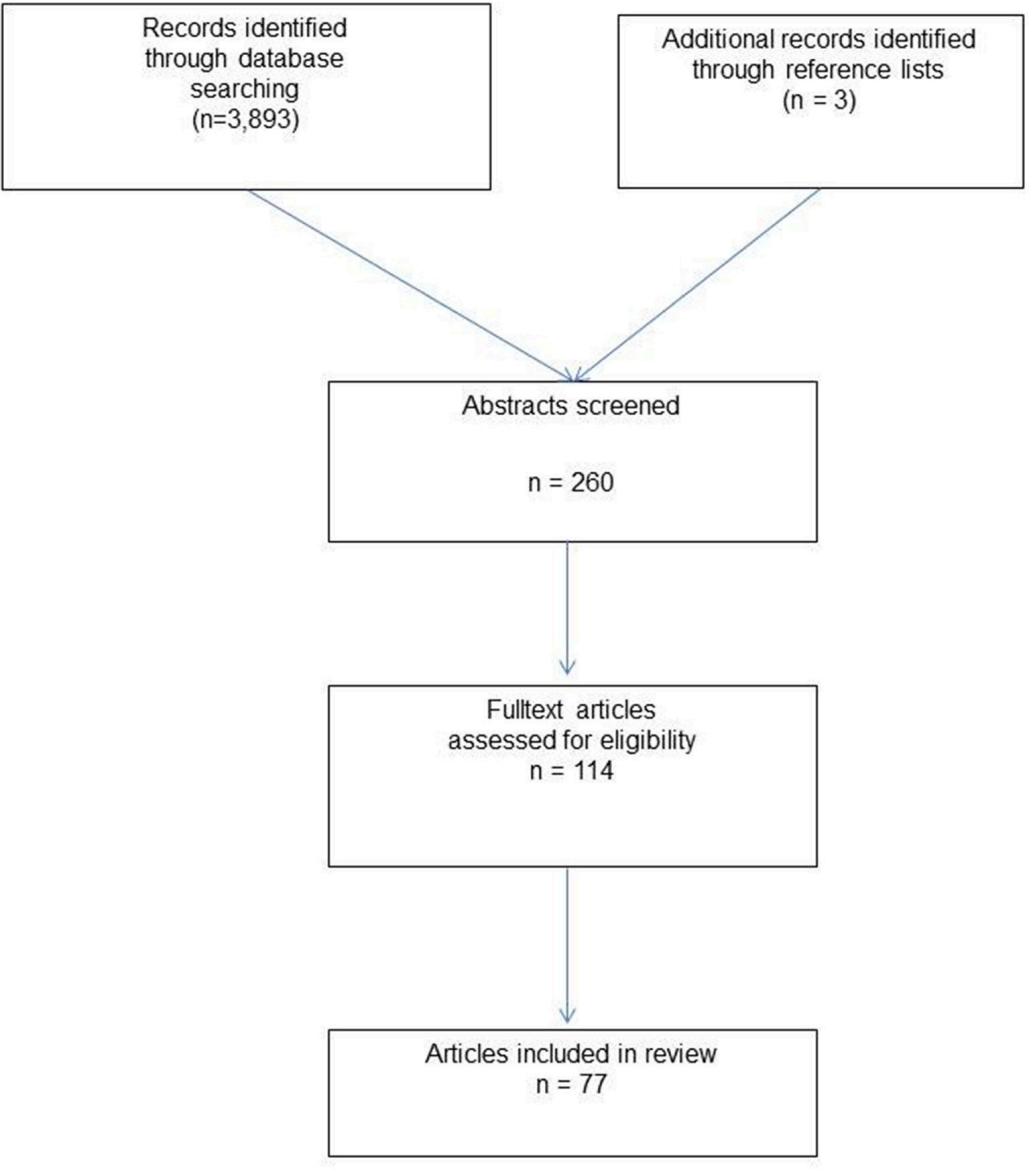
Flowchart of article inclusion.

A systematic search of the available literature was performed in June 2018 using Medline, PubMed, and the Cochrane Library, as well as a search of relevant journals and reference lists. The following search terms were used: “Sublay,” “Retro-rectus,” “Incisional hernia,” “Sublay technique,” “Rives-Stoppa technique,” “Rives-Stoppa-Wantz technique,” “Retro-rectus mesh,” “Retromuscular mesh,” Retro muscular prefascial mesh,” “Sublay and ventral hernia,” “Sublay and incisional hernia.” The abstracts of 260 publications were checked. For the present analysis 77 publications were identified as relevant to the key question (Figure 1).

## Results

### Comparison of the sublay/retro-rectus procedure with other operative techniques only in incisional hernia repair

#### Meta-analyses, systematic reviews, randomized controlled trials in incisional hernia repair

Numerous meta-analyses and systematic reviews pool primary ventral hernias and incisional hernias in their comparative evaluation of various surgical techniques (7–17). Therefore, the power of these meta-analyses and systematic reviews to answer the key question raised here is limited and they do not permit any binding statements to be made on the role of the sublay/retro-rectus technique in the repair of incisional hernia (7–17). There remain seven meta-analyses and systematic reviews that compared the outcomes of different surgical techniques for incisional hernia alone (18–26).

The same problems apply to randomized controlled trials (RCTs) that serve as the basis for the meta-analyses and systematic reviews (35–48). The 14 publications report on the findings of 11 RCTs since the results of a number of studies have been reported in several publications (35, 36, 43, 44, 46, 47). Only three RCTs (38, 45–47) were deemed suitable for answering the key question to be addressed here because the remaining studies had included a mixed patient group with primary ventral and incisional hernias (40, 43, 44), did not exclusively use the sublay/retro-rectus technique (41) or used a technique other than the sublay/retro-rectus technique (35–37, 39, 42, 48). Hence, there remains only a limited number of RCTs for evaluation of the role of the sublay/retro-rectus technique for the repair of incisional hernia. That naturally also detracts from the power of the remaining and relevant meta-analyses and systematic reviews. Below the open sublay/retro-rectus technique is compared first with the laparoscopic intraperitoneal onlay mesh (IPOM) technique, followed by comparison with the other open techniques for repair of incisional hernia, and is then evaluated. Due to the paucity of relevant RCTSs, the findings of comparative observational studies are also included in this review.

#### Laparoscopic IPOM vs. open sublay/retro-rectus technique for incisional hernia repair in meta-analysis, systematic reviews and RCTs

The meta-analysis of RCTs by Chalabi (24) included five studies (37, 40, 41, 45–47). The authors came to the conclusion that the short- and long-term outcomes of laparoscopic and open abdominal wall hernia repairs are equivalent: both techniques are safe and credible and the outcomes are very comparable (24). One aspect of this meta-analysis that must be criticized is that it contained one RCT with ventral hernia repair (40) and two RCTs with open surgical techniques other than the sublay/retro-rectus technique (37, 41). Hence the power of that meta-analysis must be greatly put into perspective.

Another meta-analysis that compared laparoscopic IPOM vs. open repair of incisional hernias by Awaiz (21–23) concluded after making an amendment that a statistically significant reduction in wound complications was noted with laparoscopic surgery compared to the open repair based on six studies (37–39, 41, 45, 46). Here, too, it must be pointed out that only in three RCTs was the open comparison group based on the sublay/retro-rectus technique (38, 45, 46).

Likewise, the last published meta-analysis of comparison of the laparoscopic vs. open repair by Dietz (26), which included only three (38, 45, 46) of nine RCTs with sublay/retro-rectus technique as open technique, identified comparative reoperation, complication and recurrence rates (26). Here only a total of 170 laparoscopic IPOM operations were compared with 181 open sublay/retro-rectus repairs.

Table 1 features the results of RCTs comparing the laparoscopic IPOM with only the open sublay/retro-rectus technique. This does not give a clear picture. One RCT showed a highly significant difference in the surgical site infection rate to the advantage of the laparoscopic IPOM (46), whereas the other two RCTs reported more postoperative complications for the laparoscopic IPOM (38, 45). No significant differences were discerned in the recurrence rates or the hospital stay (45, 46).

**Table 1:** Results of RCTs comparing laparoscopic IPOM vs. open sublay/retro-rectus technique in incisional hernia repair.

**Authors**	**Patients**	**Hernia type**	**Inclusion/exclusion**	**Postoperative complications**	**Recurrence**	**Hospital stay**
Navarra et al. (38)	Laparoscopic IPOM *n* = 12 Sublay *n* = 12	Incisional	Primary incisional only	Laparoscopic IPOM 16.6% Sublay 8.3% *p* = 0.71	–	Laparoscopic IPOM 5.7d (range 1–13d) Sublay 10.0d (range 5–19d)
Eker et al. (45)	Laparoscopic IPOM *n* = 94 Sublay *n* = 100	Incisional	Recurrent incisional hernias included	Laparoscopic IPOM 37.0% Sublay 26.0% *p* = 0.013	Laparoscopic IPOM 18% Sublay 14% *p* = 0.30	Laparoscopic IPOM 3d (range 2–4 d) Sublay 3d (range 2–5 d) *p* = 0.50
Rogmark et al. (46, 47)	Laparoscopic IPOM *n* = 64 Sublay *n* = 69	Incisional	Primary incisional or recurrent incisional without previous mesh	Laparoscopic IPOM 57% Sublay 60% *p* = 0.273 Surgical site infection Laparoscopic IPOM 1.6% Sublay 23.2% *p* < 0.001	1-year FU Laparoscopic IPOM 8.2% Sublay 1.6% *p* < 0.112	Laparoscopic IPOM 2d (range 1.5–3d) Sublay 2d (range 1–3d) *p* < 0.861

Hence, based on the available meta-analyses, systematic reviews and RCTs it is not possible to make any clear statement about which surgical technique has advantages when comparing laparoscopic IPOM vs. open sublay/retro-rectus repair. Therefore, the findings of comparative observational studies are included additionally in this review when seeking to answer the key question.

#### Laparoscopic IPOM vs. sublay/retro-rectus incisional hernia repair in comparative observational studies

In a registry-based, propensity score-matched comparison of laparoscopic IPOM and sublay/retro-rectus incisional hernia repairs, 3,965 matched pairs were created and compared. The comparison revealed disadvantages for the open sublay/retro-rectus technique regarding postoperative surgical complications, complication-related reoperations and postoperative general complications. The majority of surgical postoperative complications were surgical site occurrences. Laparoscopic IPOM had disadvantages in terms of intraoperative complications, mainly bleeding and bowel injuries. No significant differences were observed in the recurrence and pain rates at 1-year follow-up (Köckerling et al., in review).

#### Comparison of suture vs. mesh sublay/retro-rectus incisional hernia repair in meta-analyses, systematic reviews and RCTs

One meta-analysis of comparison of suture vs. mesh repair by Mathes (14) included only two RCTs for incisional hernias and sublay/retro-rectus repair (35, 36, 42), with one RCT reporting different follow-up intervals (35, 36). The results are summarized in Table 2. The publications included in the RCTs showed a significantly lower recurrence rate following sublay/retro-rectus mesh repair compared with suture repair of incisional hernias.

**Table 2:** RCTs comparing suture vs. mesh sublay/retro-rectus technique in incisional hernia repair.

**Authors**	**Patients**	**Hernia type**	**Inclusion/Exclusion**	**Intervention**	**Control**	**Outcome**
Luijendijk et al.(35)	Suture repair *n* = 97 Mesh sublay repair *n* = 84	Incisional	Primary and first recurrent incisional hernia	Suture repair	Mesh sublay repair	3-year follow-up Suture repair: Recurrence rate 47% Sublay repair 27% *p* = 0.005
Burger et al. (36)	Suture repair *n* = 97 Mesh sublay repair *n* = 84	Incisional	Primary and first recurrent incisional hernia	Suture repair	Mesh sublay repair	10-year follow-up Suture repair: Recurrence rate 63% Sublay repair: Recurrence rate 32% *p* < 0.001
Venclauskas et al. (42)	Suture repair *n* = 54 Mesh sublay repair *n* = 50	Incisional	Primary incisional or recurrent incisional without previous mesh	Suture repair	Mesh sublay repair	1-year follow-up Suture repair: Recurrence rate 22.2% Sublay repair: 2.0% *p* = 0.002

#### Comparison of onlay vs. sublay/retro-rectus incisional hernia repair in meta-analyses, systematic reviews, RCTS and comparative observational studies

The meta-analysis by Timmermans (25) compared the sublay /retro-rectus with the onlay technique only for incisional hernias. For that meta-analysis only two RCTs (42, 44) were identified, with one RCT also featuring primary abdominal wall hernias (44). The other studies were one prospective (49) and seven retrospective comparative studies (50–56). The meta-analysis then compared 775 onlay operations with 1,173 sublay/retro-rectus operations (25). A trend was observed for incisional hernia recurrence in favor of the sublay repair (OR 2.41; 95% CI [0.99–5.88] *p* = 0.05) (25). Surgical site infection occurred significantly less often after sublay/retro-rectus repair (OR = 2.42; 95% CI [1.02–5.74]; *p* = 0.05) (25). The results of the meta-analysis were also confirmed by the three RCTs available in the meantime comparing sublay/retro-rectus vs. onlay incisional hernia repair (42, 57, 58) (Table 3). Here, too, significantly more wound complications as well as a trend toward higher recurrence rates were identified for onlay repair of incisional hernias.

**Table 3:** RCTs comparing sublay/retro-rectus vs. onlay mesh repair technique in incisional hernia repair.

**Authors**	**Patients**	**Hernia type**	**Inclusion/Exclusion**	**Postoperative complications**	**Recurrence**	**Hospital stay**
Venclauskas et al. (42)	Sublay *n* = 50 Onlay *n* = 50	Incisional	No recurrent incisional hernia	Wound complications: Sublay 24.0% Onlay 49.1% *p* < 0.004 Seroma: Sublay 24.0% Onlay 45.6% *p* < 0.001	1-year follow-up Sublay 2.0% Onlay 10.5% *p* = 0.077	Sublay 5.5 ± 1.6 Onlay 5.9 ± 2.3
Demetrashvili et al. (57)	Sublay *n* = 90 Onlay *n* = 90	Incisional	No recurrent incisional hernia	Wound complications: Sublay 22.1% Onlay 50.0% *p* < 0.001 Seroma: Sublay 16.9% Onlay 41.0% *p* = 0.0013	Sublay 2.6% Onlay 5.1% n.s.	–
Sevinc et al. (58)	Sublay *n* = 50 Onlay *n* = 50	Incisional	No recurrent incisional hernia	Wound complications: Sublay 8.0% Onlay 24.0% *p* = 0.029	Median follow-up: 37.1 months Sublay 2% Onlay 6% *p* = 0.307	Sublay 3.52 ± 2.6 Onlay 3.36 ± 1.9 *p* = 0.734

In a nationwide prospective study of the Danish Hernia Database of outcomes after elective incisional hernia repair, the sublay/retro-rectus mesh position resulted in a significantly lower risk for recurrence operations (cumulative risk 12.1%) compared with onlay mesh position (16.1%) and intraperitoneal mesh position (21.2%) (*p* = 0.03) (59).

#### Comparison of underlay/intraperitoneal vs. sublay/retro-rectus mesh incisional hernia repair in comparative observational studies

No RCTs are available for comparison of the open IPOM technique/underlay technique vs. the open sublay/retro-rectus technique. The existing meta-analyses also include primary ventral hernias (15–17). Besides, one RCT for that comparison is not available. Therefore, recourse had to be had to comparative observational studies.

It has already been pointed out above that in the Danish registry study the recurrence rate at 21.2% was markedly higher for the open intraperitoneal onlay mesh technique or underlay technique compared with the sublay/retro-rectus technique (59).

### Details of the sublay/retro-rectus incisional hernia repair technique

**Figure 2:**
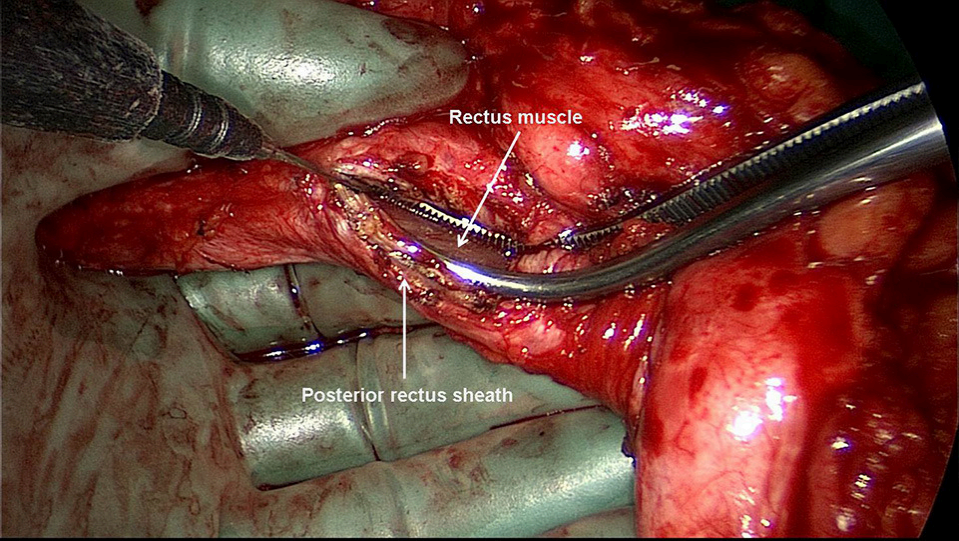
The posterior rectus sheath is opened near the linea alba and the posterior aspect of the rectus muscle exposed.

**Figure 3:**
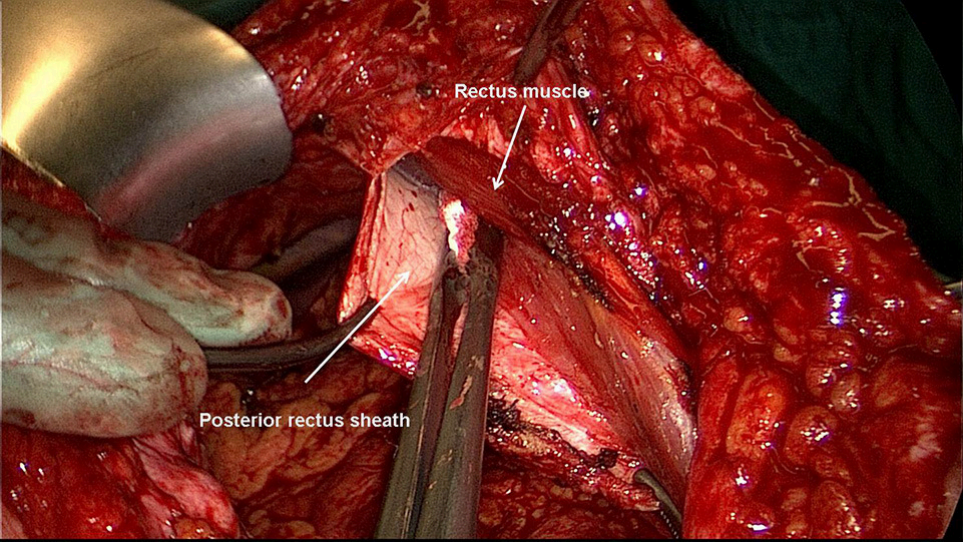
The space between the posterior rectus sheath and the rectus muscle is developed using a combination of blunt and sharp dissection.

**Figure 4:**
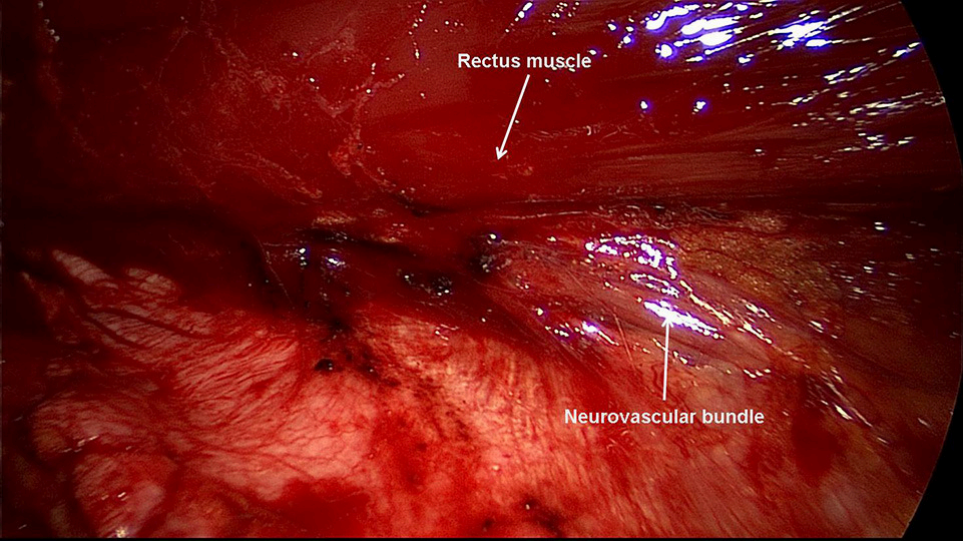
Careful identification and preservation of the intercostal nerves and vessels are critical.

**Figure 5:**
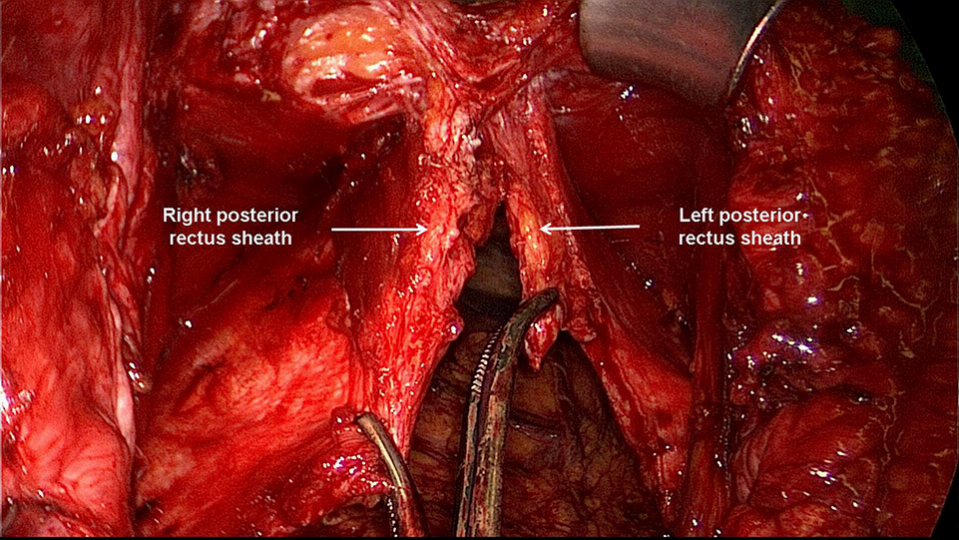
The retromuscular plane can be extended cephalad to the retroxiphoid and retrosternal areas. Typical finding before transection of the right and left posterior rectus sheath.

**Figure 6:**
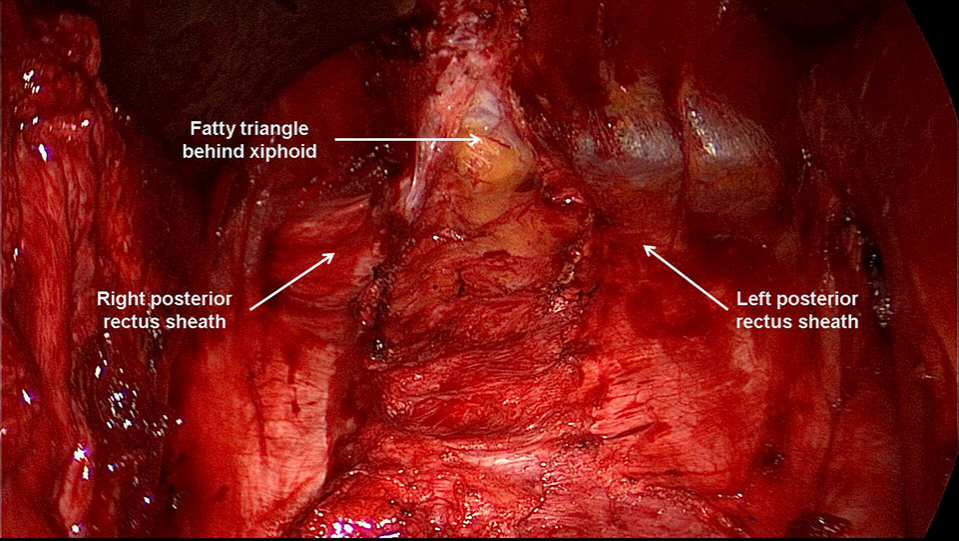
Typical finding after transection of the right and left posterior rectus sheaths and opening of the space between fatty triangle and xiphoid.

**Figure 7:**
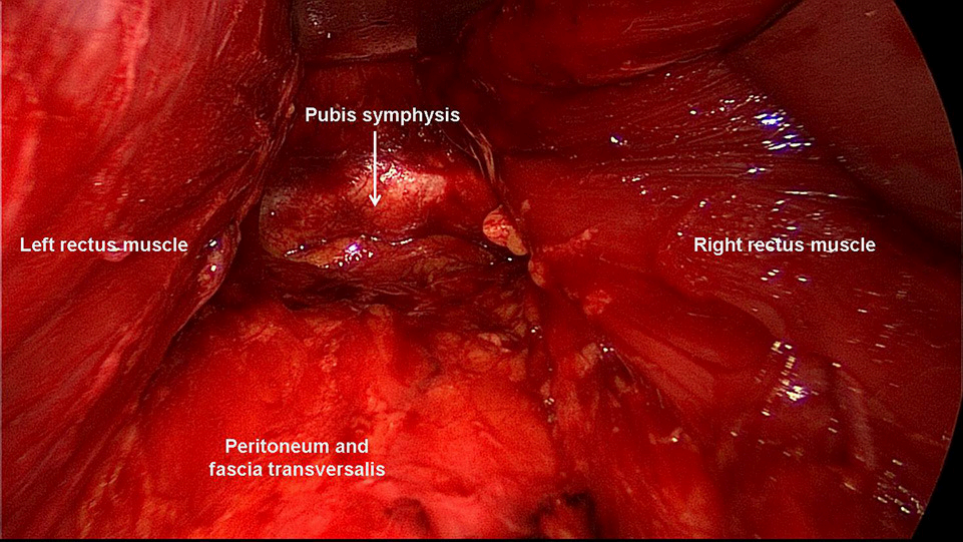
Inferiorly, the space of Retzius is entered to expose the pubis symphysis. Below the arcuate line, the posterior layer only includes peritoneum and transversalis fascia.

**Figure 8:**
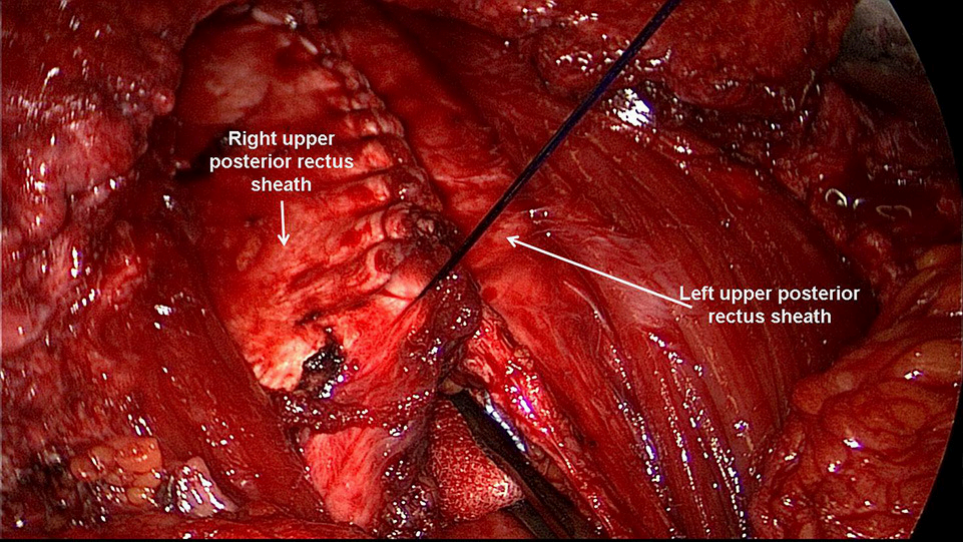
Closure of the posterior rectus sheath cranially.

**Figure 9:**
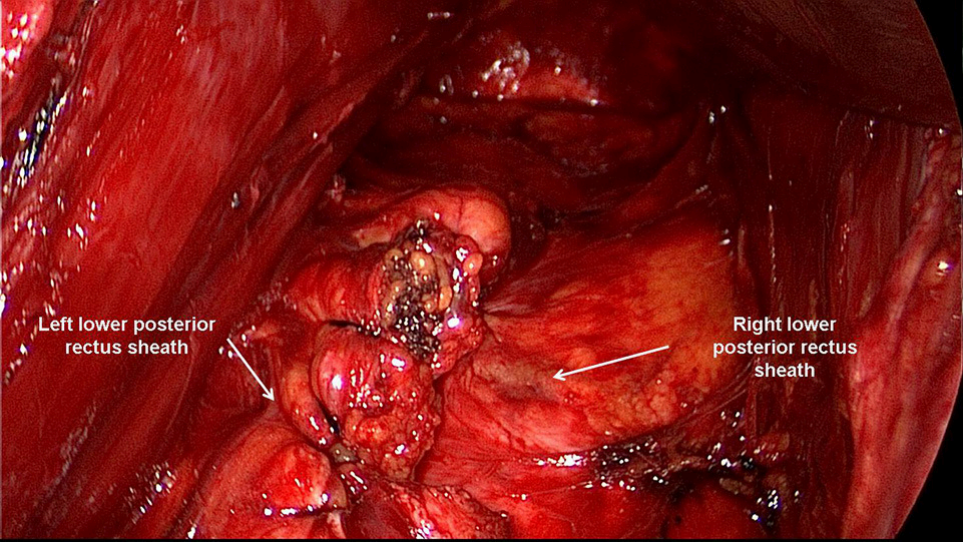
Closure of the posterior rectus sheath caudally.

**Figure 10:**
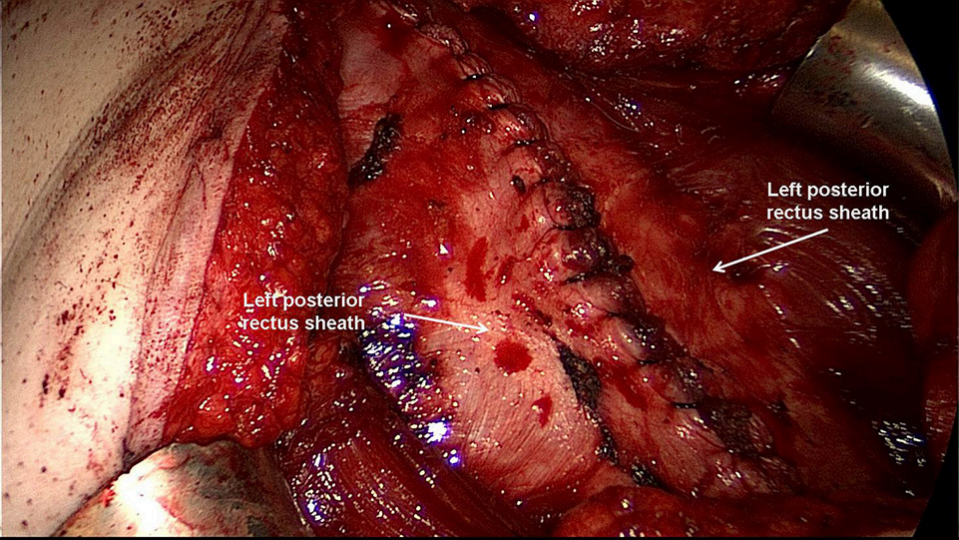
Complete reconstruction of the posterior rectus sheath.

**Figure 11:**
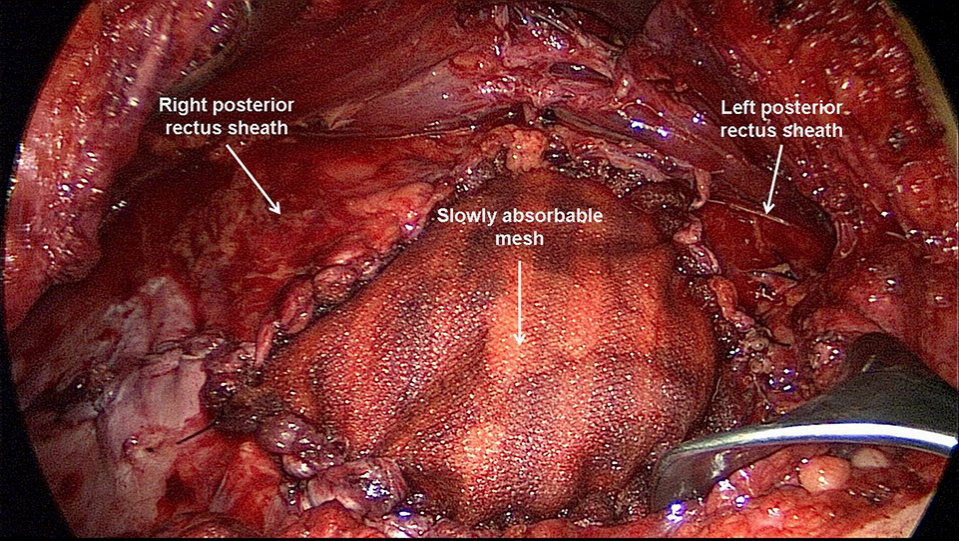
Closure of a remaining defect in the posterior rectus sheath by a slowly absorbable mesh (Phasix ST).

**Figure 12:**
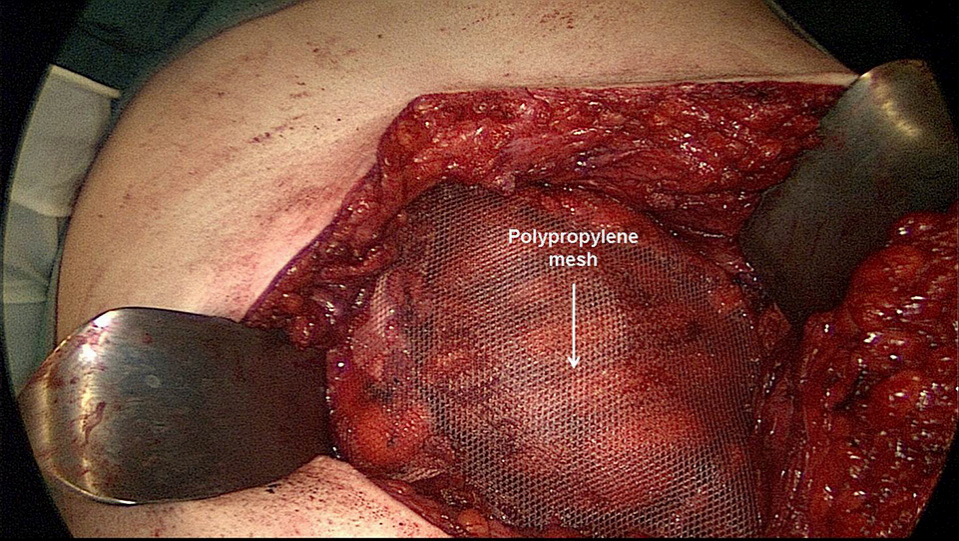
The non-absorbable mesh (TiMesh strong) is placed between the rectus abdominis muscle, the ribs and xiphoid process and the reconstructed posterior rectus sheath.

**Figure 13:**
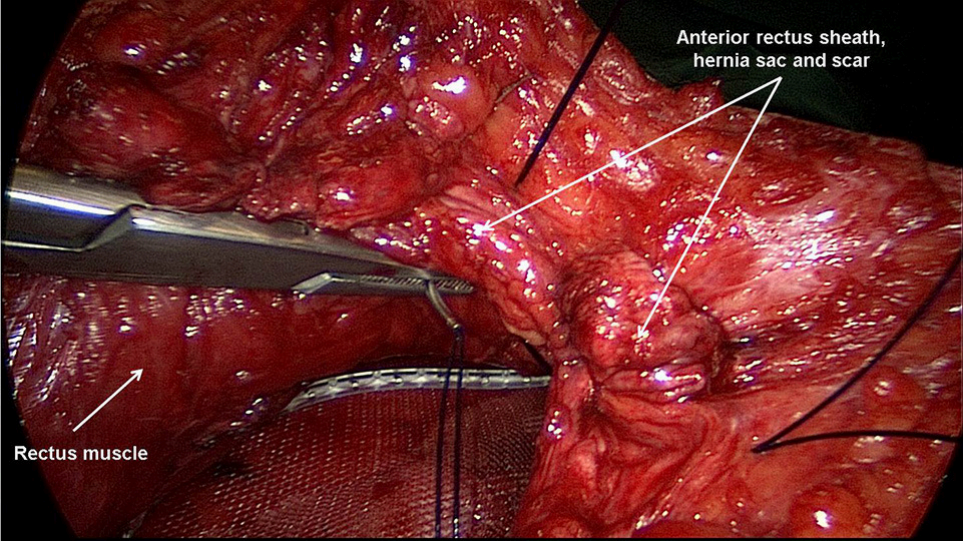
Reconstruction of the linea alba by suturing together the anterior rectus sheath, hernia scar and the remaining hernia sac over the mesh.

The most important technical steps of sublay/retro-rectus repair of incisional hernias are as follows (60–65):

- Excision of scar and partial hernia sac, adhesiolysis (60–65)
  For avoidance of wound complications the thinned out portion of skin with the part of the hernia scar remaining in this area and the scar are elliptically excised. Excision of the umbilicus is also recommended. Further dissection between the skin/subcutaneous tissue and myofascial abdominal wall should be avoided. The remaining parts of the scarred hernia sac margins should be preserved and are later included in the suture of the anterior rectus sheath. Via the opened hernia sac adhesiolysis of intestinal loops and parts of the greater omentum is then carried out.
- Incision of the posterior rectus sheath and creation of the retromuscular, prefascial space (60–65)
  The position of the mesh in the space between the rectus abdominis muscle and the posterior rectus sheath requires opening of the rectus sheath. The posterior rectus sheath is opened near the linea alba to enter the retromuscular space and expose the posterior aspect of the rectus muscle (Figure 2). The space is developed using a combination of blunt and sharp dissection (Figure 3). The lateral extent of this dissection is the linea semilunaris, the junction between the posterior and anterior rectus sheaths. Careful identification and preservation of the intercostal nerves and vessels is critical to maintaining an innervated functional abdominal wall (Figure 4). The retromuscular plane can be extended cephalad to the retroxiphoid and retrosternal areas (Figures 5, 6). Inferiorly, the space of Retzius is entered to expose the pubis symphysis and both Cooper's ligaments (Figure 7). Since this area is below the arcuate line, posterior layer includes peritoneum and transversalis fascia only (Figure 7).
- Closure of the posterior rectus sheath (60–65)
  In most cases, suture of the posterior rectus sheath's margins can be achieved when correctly and widely freed (Figures 8–10). When closure of the posterior rectus sheath is not possible, the remaining defect can be closed by a slowly absorbable mesh (Figure 11).
- Placement of the mesh and fixation (60–65)
  The upper part of the mesh is placed between the rectus abdominis muscle, the ribs and xiphoid process and the reconstructed posterior rectus sheath. The lower part of the mesh is fixed to Cooper's ligament. Centrally, the mesh is placed on the retromuscular space limited by the linea semilunaris (Figure 12). The mesh can be fixed circumferentially with full-thickness, transabdominal sutures using a Reverdin needle or by single sutures to the posterior rectus sheath. Finally, the linea alba is reconstructed by suturing together the anterior rectus shealt, hernia scar and the remaining hernia sac over the mesh (Figure 13).
- Comparison of lightweight vs. standard mesh in sublay/retro-rectus repair of incisional hernias
  One meta-analysis (66) compared the results of the sublay/retro-rectus repair of incisional hernias based on a single RCT (67), three prospective (68–70) and one retrospective (71) study. The authors concluded that the use of lightweight mesh in open sublay/retro-rectus repair seems to be associated with less chronic pain, and with no increase in recurrence or in other postoperative complications (66).
  Another RCT compared for incisional hernias the sublay/retro-rectus technique with a lightweight polypropylene mesh and a partially absorbable polypropylene mesh (72). With a total of 80 randomized patients no significant difference was observed between these two groups (72).
- Comparison of self-adhering meshes with suture fixation in sublay/retro-rectus incisional hernia repair
  In a comparative study 12 patients with transfacially sutured mesh and 14 patients with self-adhering mesh in sublay/retro-rectus incisional hernia repair were followed up for at least 12 months. The results show low rates of surgical site occurrences, recurrences and significantly less acute pain with self-adhering mesh (73).
  In a prospective comparative study with 50 patients comparing self-adhering, with suture fixed, meshes in sublay/retro-rectus incisional hernia repair, postoperative pain in the first 48 h was less in the self-adhering mesh group (74).
- Comparison of fibrin glue vs. transfascial suture mesh fixation
  In a comparative study of open abdominal wall reconstruction with retromuscular mesh fixation using fibrin glue vs. transfascial sutures the probability of reporting pain at 6-month follow-up was significantly higher in the transfascial suture group (OR 12.29, 95% CI [1,26–120.35]; *p* = 0.031) (75). No hernia recurrences were noted in either group with a mean follow-up of 390 ± 330 days (75).
- Comparison of drain vs. no drain placement
  In a registry-based comparison of drain placement vs. no drain placement of the retromuscular ventral hernia repair surgical drains do not increase the risk of surgical infection complications and may be protective against some surgical site occurrences, such as seroma formation (76).

## Discussion

There are considerable limitations when engaging in critical analysis of the literature available for evaluation of the sublay/retro-rectus technique for repair of incisional hernia. Several meta-analyses, systematic reviews and RCTs report on a pooled patient group with primary (umbilical hernias, epigastric hernias) and secondary (incisional hernias) abdominal wall hernias (7–17, 40, 43, 44) despite the fact that there are significant differences in the outcomes of primary and secondary abdominal wall hernias (30–33). Hence, these findings are only of limited value. Furthermore, the open incisional hernia repair group also included other techniques in addition to the sublay/retro-rectus technique (35–39, 41, 42, 48). As such, only a very limited number of studies are available for evaluation of the sublay/retro-rectus technique in repair of incisional hernia.

When comparing open sublay/retro-rectus repairs of incisional hernias it is not possible to base a reliable evaluation of these operations on the existing meta-analyses, systematic reviews or RCTs because of the contradictory nature of the data (38, 45, 46). Only one registry-based, prospective observational study with a relatively large case number demonstrated clear advantages for the laparoscopic IPOM in the early postoperative course thanks to a significantly low postoperative complication rate, in particular in respect of the surgical site occurrences, complication-related reoperations and the general postoperative complications. Disadvantages of laparoscopic IPOM related to intraoperative complications. No differences were identified in the pain and recurrence rates at 1-year follow-up (Köckerling et al., in review).

However, the open sublay/retro-rectus technique has been found to have advantages over the other incisional hernia open repair techniques (14, 25, 58). In comparison with the open suture technique, onlay and underlay or intraperitoneal onlay technique, consistently lower recurrence rates have been identified for the sublay/retro-rectus technique. Likewise, the surgical site infection rate is lower after sublay/retro-rectus repair of incisional hernias than after the onlay technique (45).

Although there are much less data available for evaluation of the sublay/retro-rectus technique for repair of incisional hernia than suggested by the myriad meta-analyses and systematic reviews, the sublay/retro-rectus technique appears to have advantages over the other open techniques. But it appears to have more disadvantages compared with laparoscopic IPOM.

However, a precise analysis of the existing literature clearly demonstrates that further studies are urgently needed to evaluate the role of the sublay/retro-rectus technique in repair of incisional hernia. To that effect, it is of paramount importance to focus on a single hernia entity and on two comparative surgical techniques. To evaluate the outcome it would also appear important to include here all details of the sublay/retro-rectus surgical technique with its different variants of meshes, fixation techniques and drain placement (60–76). Therefore, further RCTs using a standardized technique and restricted to incisional hernias should be carried out for comparison with both the laparoscopic IPOM and with other open techniques.

## Author contributions

FK literature search, literature analyses, publication concept, publication draft. HS and CS-P literature search, literature analyses, publication concept, critical review of the publication draft.

### Conflict of interest statement

The authors declare that the research was conducted in the absence of any commercial or financial relationships that could be construed as a potential conflict of interest.
